# Barriers and enablers to the implementation of protocol-based imaging in pancreatic cancer: A qualitative study using the theoretical domains framework

**DOI:** 10.1371/journal.pone.0243312

**Published:** 2020-12-17

**Authors:** Ashika D. Maharaj, Sue M. Evans, John R. Zalcberg, Liane J. Ioannou, Marnie Graco, Daniel Croagh, Charles H. C. Pilgrim, Theresa Dodson, David Goldstein, Jennifer Philip, James G. Kench, Neil D. Merrett, Rachel E. Neale, Kate White, Peter Evans, Trevor Leong, Sally E. Green

**Affiliations:** 1 School of Public Health and Preventive Medicine, Monash University, Melbourne, Australia; 2 Cancer Council Victoria, Melbourne, Victoria, Australia; 3 Alfred Health, Melbourne, Victoria, Australia; 4 Monash Health, Clayton, Victoria, Australia; 5 Prince of Wales Clinical School, University of New South Wales, Randwick, New South Wales, Australia; 6 St Vincent’s Hospital Melbourne, Melbourne, Victoria, Australia; 7 University of Melbourne, Melbourne, Victoria, Australia; 8 Royal Prince Alfred Hospital, Camperdown, New South Wales, Australia; 9 School of Medicine, Western Sydney University, Penrith South, New South Wales, Australia; 10 QIMR Berghofer Medical Research Institute, Herston, Queensland, Australia; 11 Sydney Nursing School, University of Sydney, Sydney, New South Wales, Australia; 12 Peninsula Health, Frankston, Victoria, Australia; 13 Peter MacCallum Cancer Centre, Melbourne, Victoria, Australia; MD Anderson Cancer Center, UNITED STATES

## Abstract

**Background:**

Accurate pre-operative imaging plays a vital role in patient selection for surgery and in allocating stage-appropriate therapies to patients diagnosed with pancreatic cancer (PC). This study aims to: (1) understand the current diagnosis and staging practices for PC; and (2) explore the factors (barriers and enablers) that influence the use of a pancreatic protocol computed tomography (PPCT) or magnetic resonance imaging (MRI) to confirm diagnosis and/or accurately stage PC.

**Methods:**

Semi-structured interviews were conducted with radiologists, surgeons, gastroenterologists, medical and radiation oncologists from the states of New South Wales (NSW) and Victoria, Australia. Interviews were conducted either in person or via video conferencing. All interviews were recorded, transcribed verbatim, de-identified and data were thematically coded according to the 12 domains explored within the Theoretical Domains Framework (TDF). Common belief statements were generated to compare the variation between participant responses.

**Findings:**

In total, 21 clinicians (5 radiologists, 10 surgeons, 2 gastroenterologists, 4 medical and radiation oncologists) were interviewed over a four-month-period. Belief statements relevant to the TDF domains were generated. Across the 11 relevant domains, 20 themes and 30 specific beliefs were identified. All TDF domains, with the exception of *social influences* were identified by participants as relevant to protocol-based imaging using either a PPCT or MRI, with the domains of *knowledge*, *skills* and *environmental context and resources* being offered by most participants as being relevant in influencing their decisions.

**Conclusions:**

To maximise outcomes and personalise therapy it is imperative that diagnosis and staging investigations using the most appropriate imaging modalities are conducted in a timely, efficient and effective manner. The results provide an understanding of specialists’ opinion and behaviour in relation to a PPCT or MRI and should be used to inform the design of future interventions to improve compliance with this practice.

## Introduction

Accurate pre-operative imaging plays a vital role in patient selection for surgery and in allocating stage-appropriate therapies to patients diagnosed with pancreatic cancer (PC) [1]. The recommended method of assessing operability is to use a high-quality multi-phase computed tomography (CT) scan that examines the abdominal area in the arterial and portal venous phase. Such a CT scan can determine the proximity of the tumour to major vascular structures and the presence of locally advanced disease or intra and extra-abdominal metastases [2, 3]. In 2012, the Society of Abdominal Radiology and the American Pancreatic Association released a consensus statement describing a standardised reporting template for the accurate staging of PC to improve disease management. This statement was authored by a multi-institutional group of experts comprising radiologists, gastroenterologists, and hepatopancreatobiliary surgeons [4]. For accurate disease staging, it was recommended that all patients with no obvious metastatic disease or local invasion at initial routine CT, undergo a repeat examination with a dedicated pancreas protocol multiphase computed tomography (PPCT) prior to endoscopy, biliary stenting or invasive tissue sampling [4–6].

More recently, evidence-based clinical practice guidelines recommend that PPCT be used to assess the extent of the disease across all stages of PC [7–9]. Where there is clinical suspicion of PC, a PPCT is also considered to be the primary imaging modality to diagnose and stage the extent of the disease within one single session [10, 11]. The pancreas is anatomically intertwined with critical vascular structures: specifically, the celiac artery, hepatic artery, superior mesenteric artery, portal vein and the superior mesenteric vein. A PPCT evaluation of the vascular involvement is highly predictive of the extent of vascular involvement which determines operability and overall survival [6]. In addition, a PPCT is also required for accurate planning and delivery of targeted radiotherapy, which requires clear delineation of the tumour in relation to normal pancreatic parenchyma and surrounding normal structures.

Magnetic resonance imaging (MRI) is considered by some to be equivalent to a CT in detecting and staging PC [12]. However, recent evidence recommends the addition of MRI as an adjunct to detect the presence of liver metastases, rather than a replacement for PPCT [3]. The liver is the most common organ affected by metastasis, and establishing the accurate extent of liver disease can help avoid futile attempts at surgical resection [13]. It has been suggested that all patients with PC deemed resectable should undergo liver MRI to complement CT evaluation, but this is not always feasible due to limited resources and access to MRI [3].

A set of quality indicators for PC was developed by Australian clinicians in 2018 and compliance with these indicators is reported by the Upper Gastrointestinal Cancer Registry (UGICR) [14, 15]. Preliminary analysis of registry data found that 29% (n = 64/224) of patients with potentially resectable and 43% (n = 54/125) of patients with locally advanced disease (without metastases) did not undergo either a documented PPCT or MRI. In a disease where surgery is complex but remains the best chance for long-term survival in patients with localised disease, incomplete or sub optimal pre-operative staging can result in adverse outcomes, such as planned procedures and surgery being abandoned intraoperatively or a margin-positive resection, resulting in poorer survival [16].

Given the likely benefits of PPCT and MRI, and the evidence for variation in these practices, it is important to understand the reasons why patients with PC may or may not be receiving this care. Here we explore the barriers and enablers to the implementation of a PPCT or MRI for diagnosis and/or staging of PC. In so doing, we will inform tailored knowledge translation and quality improvement interventions which address modifiable barriers and enhance enablers of PPCT and MRI use.

This study aims to: (1) understand the current diagnosis and staging practices for PC; and (2) explore the factors (barriers and enablers) that influence the use of a PPCT or MRI to confirm diagnosis and/or accurately stage PC.

## Methods

**Fig 1:**
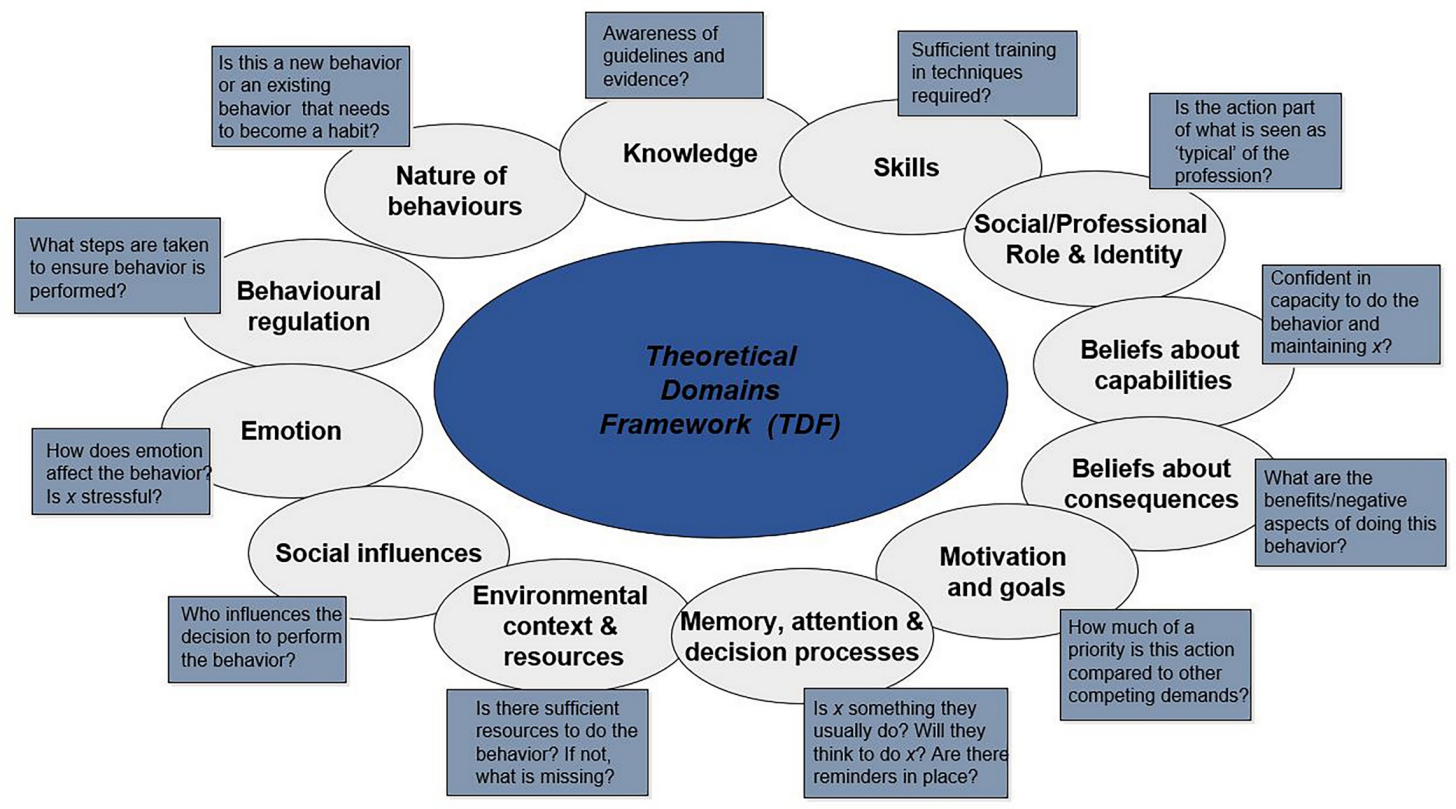
Theoretical domains framework [19].

This study is underpinned by the theoretical domains framework (TDF). The TDF was developed through international collaboration between behavioral scientists and implementation researchers. The framework consolidates theories relevant to behavioral and psychological processes determining the influences on healthcare practitioners’ behavior. Constructs from these theories are grouped into domains (Fig 1) and provide a theoretical lens to identify the determinants of behavior [17–19].

### Step one: Describing ideal practice—focus group

A focus group was convened comprising clinical experts involved in the treatment and management of PC to understand the steps involved in the use of PPCT and MRI in a clinical setting. Following a systematic approach for using the TDF to implement evidence-based interventions into practice [20], the group considered ‘*who needs to do what differently*, *when*, *where and how’* and developed a decision tree which clearly articulated the clinical decisions and processes involved in appropriate execution of PPCT and MRI. This decision tree formed the reference, or ‘ideal practice’, of the interview questions; these were grouped into domains according to the TDF.

### Step two: Understanding the barriers to and enablers of ideal practice- semi-structured interviews

Medical specialists involved in the treatment of patients with PC (radiologists, surgeons, medical and radiation oncologists and gastroenterologists) from public and private hospitals, within metropolitan and regional areas, across the states of New South Wales (NSW) and Victoria were invited to participate in semi-structured interviews underpinned by the TDF. A stratified purposeful sampling strategy was used to recruit participants, followed by snowball sampling [21]. This initially involved inviting by email clinicians contributing to the UGICR and clinicians identified as specialising in PC on hospital websites [15]. In addition, those interviewed were asked to nominate other specialists for researchers to contact. Participants who opted-in to the study provided consent through email. Non-responders were sent a reminder email.

An interview guide was developed exploring factors influencing the implementation of protocol-based imaging (CTs and MRIs) in the management of all patients diagnosed with PC. Open-ended questions were asked to cover each domain of the TDF. The interview schedule was initially piloted with two surgeons (DC and CHCP) and a nurse specialist (TD), following which it underwent further refinement. The interview schedule is provided as S1 File.

Two researchers (ADM—lead interviewer and SEG) conducted interviews either in person or using Zoom video conferencing (https://zoom.us/). With participants’ consent, interviews were recorded on video and audio using Zoom conferencing software, transcribed verbatim, de-identified and imported into NVIVO 12.0 Plus qualitative software (QSR International Pty Ltd. Version 12, 2018) for analysis.

### Analysis

Interview transcripts were thematically coded according to the 12 domains of the TDF. Overarching themes perceived to influence the implementation of protocol-based imaging and associated belief statements were then generated. A belief statement was defined as “a collection of responses with a similar underlying belief” that determined the relevance and role of the domain in influencing the behaviours around protocol-based imaging [17, 22].

A random sample of transcripts (n = 3, 10%) were analysed independently by two researchers, who then met to discuss the generation of themes and coding guidelines. Any discrepancies were discussed with another researcher for resolution. The full summary of results was independently reviewed and presented to the same focus group as Step One above over two meetings for feedback and interpretation of findings.

### Ethics

Ethics approval was obtained from Monash University Human Research Ethics Committee (MUHREC): Project Number 19446.

## Results

### Focus group

Twelve clinical experts were invited to the initial focus group and nine participated. The focus group comprised three surgeons, two medical oncologists, a radiation oncologist, a palliative care specialist, a nurse specialist, and a nurse care coordinator. The decision tree, presented in S1 Fig, clearly articulated the clinical pathway leading to a PPCT or MRI, and the appropriate precursors and outcomes. This was agreed by the focus group as the evidence- and consensus- based ideal clinical practice.

### Interviews

A total of 49 specialists were approached across the two states over a four month period (August–December 2019) and 21 (40%) participants were interviewed. Participant characteristics are summarised in Table 1. Most were male (n = 20, 95%); 71% were from metropolitan settings, and 62% were from Victoria. The case volume of surgeons varied by state and rurality. Surgeons in the state of Victoria and in regional areas commonly saw between one and three PC cases per week, compared to five to ten PC cases seen by surgeons working in NSW and metropolitan regions. This difference is likely related to the model of centralised care in NSW. Clinicians working in other disciplines from both states reported seeing from zero to two cases per week.

**Table 1:** Participant characteristics.

			State in Australia		Area of Work		Type of Hospital	
DISCIPLINE	Invited	Included	New South Wales	Victoria	Metropolitan	Regional	Public	**Private**
Surgeon	18	10	4	6	7	3	9	1
Radiologist	7	5	1	4	4	1	4	1
Med. Oncologist	13	3	1	2	1	2	2	1
Rad. Oncologist	4	1	1	0	1	-	1	-
Gastroenterologist	7	2	1	1	2	-	2	-
**TOTAL**	**49**	**21**	8	13	15	6	18	3

### Factors influencing the implementation of a PPCT or MRI

Across the relevant domains, 20 themes and 30 specific beliefs were identified. Table 2 summarises the main belief statements, categorised according to the domains of the TDF. Each belief statement is demonstrated by representative quotes.

**Table 2:** Summary of relevant TDF domains, belief statements and representative quotes.

Implementation of a pancreatic protocol CT (PPCT) or magnetic resonance imaging (MRI) for diagnosis and/or staging			
Domain	Themes	Belief Statements	Representative Quotes
**Knowledge**	Clinical Practice Guidelines	I am aware of clinical practice guidelines	*“We’re all aware of the NCCN (National Comprehensive Cancer Network) guidelines*, *they're household names*, *fairly general too*, *and they're fairly easy to adhere to*. *They don’t influence practice too much*, *because we have our own department protocols and procedures that we regard as more important than established international guidelines” (Radiation Oncologist)*
			*“I’m aware of them*, *I don’t refer to them*. *I know they exist*.*” (Hepatopancreatobiliary (HPB) Surgeon)*
		I do not know or am not aware of clinical practice guidelines	*“All of the medical oncologists would not necessarily know of them*, *and all of the GPs definitely wouldn’t know of them” (HPB Surgeon)*
	Research	I am aware of research about PPCT or MRI	*“There has been research done on those in terms of diagnosis*. *We’ve looked more at the locally advanced and borderline resectable tumours*, *looking at what are the criteria [on PPCT or MRI] which will be used on those patients who may be suitable for neo-adjuvant therapies” (HPB Surgeon)*
		I am not aware of any research about PPCT or on MRIs	*“I haven’t actually read any of that literature in any sort of meaningful form actually since finishing my training which was quite a long time ago” (HPB Surgeon)*
	Difference between a PPCT and a MRI	A PPCT and MRI are equivalent	*“I think generally speaking they're fairly equivalent” (Radiologist)*
			*“Look there are minor strengths and weaknesses of each*, *but as a rule they're pretty equivalent*. *It's rare that once you’ve done one good quality computed tomography (CT) or MRI that you need to get the other one done as well” (Gastroenterologist)*
		There are differences between a PPCT and MRI	*“MRI is more challenging as a modality to get good quality imaging*. *For patients*, *it takes longer and they have to be more cooperative for that period*, *so it is a more challenging modality to do well*. *CT on the other hand is pretty quick*, *the techs are great*, *the patients don’t have to lie very still for very long” (Radiologist)*
			*“We believe the CT is better with better resolution and anterior image*. *Most radiologists elsewhere believe that MRI is better especially when they are not specialists because it is that feeling that in the evolution of technology MRI came after therefore its better*. *(Radiologist)*
			*“For the pancreas the CT is better*, *but if you’ve got a suspected liver lesion than the MRI is better” (HPB Surgeon)*
**Skills**	Radiology	Specialist Radiologists are critical to performing and interpreting findings	*“We need to have a radiologist or radiology practice who is well versed and skilled in these procedures*, *particularly in terms of the timing of the procedures that they perform*. *(HPB Surgeon)*
			*“The only people who have that understanding are either radiologists who regularly have an interest and expertise and experience in assessing CT pancreas and some HPB surgeons*.*” (Radiologist)*
	Other	Apart from radiology, other skills are necessary for diagnosis and staging of pancreatic cancer (PC)	*“Ultimately the formal reporting of the study is solely the domain of the radiologist*. *But in terms of establishing the staging*, *it would be discussed in the MDM setting and then you would have input from surgeons*, *gastroenterologists*, *radiologists*, *oncologists and also the pathologists where relevant*.*” (Radiologist)*
**Social/Professional Role and Identity**	Role for diagnosing and staging	The pathway to diagnosing and staging is complex	*” General surgeons are involved*, *as opposed to HPB surgeons*, *particularly in the country*. *Gastroenterologists are involved because the patients often present with jaundice and potentially oncologists are involved because they may get a referral with a suspicion of pancreatic cancer*. *Then it would be emergency department doctors*, *as a large proportion present acutely with jaundice and they would first be seen in the emergency department*. *And then general practitioners (GPs) will also get patients who present with weight loss*, *back pain*, *and jaundice*, *and finally endocrinologists sometimes identify these patients*, *if they have new onset diabetes or diabetes that is more difficult to control they might raise their suspicion and diagnose the pancreatic lesion” (HPB Surgeon)*
**Beliefs about Capabilities**	Challenges	PPCT or MRI is often not the primary path for diagnosis	*“The problem is patients often have symptoms for quite some time*, *and at a primary care level*, *some end up waiting a long time*. *They often are referred to a gastroenterologist; it is a long waiting time to see a gastroenterologist who then often end up doing an endoscopy and a colonoscopy*. *Then the penny drops that there is something more in about 10% of cases*. *That’s a bit irritating from our point of view*, *when the patient’s had symptoms there*, *it would’ve been easier just to do the decent CT scan*.*” (HPB Surgeon)*
	Imaging	PC can be identified using a PPCT or MRI	*“The obvious ones are obvious*, *and the difficult ones are difficult*. *We would see most cases*, *but I can certainly think offhand of a number of cases where there was a known pancreatic cancer that we couldn’t identify*. *So there is certainly cases we don’t see” (Radiologist)*
			*“In 70 or 80% of the cases you’re going to get the diagnosis with a good quality CT but you have to remain mindful of the patients who have presented with symptoms of concern such as weight loss*, *new onset diabetes or abdominal pain*. *You have to be careful not to rely solely on the CT and so therefore liberal use of endoscopic ultrasound may be appropriate*.*” (HPB Surgeon)*
		The quality and interpretation play a large role in my confidence to diagnose PC	*“A good CT is better than a bad MRI*.*” (HPB Surgeon)*.
			*Virtually all of the major HPB and pancreatic cancer cases need further imaging—ever have somebody who has had all the diagnostic imaging work done*, *I would say never*. *The only time that that would ever happen is if it's been referred to me directly (HPB Surgeon)*
			*I think pancreas cancer is much harder to diagnose in the undefined patient where you're doing a general CT and your index of suspicion is not very high*, *and it can be a very subtle and an easily missed finding*.*” (Radiologist)*
**Beliefs about consequences**	Benefits	PPCT or MRI are the best available imaging modalities for diagnosing and staging of PC	*“CT imaging is our cornerstone*, *and having good quality CT imaging is essential*, *and MRI can sometimes add important additional information” (Rad Oncologist)*
			*“If you ever get referred to a radiology department for a CT with query pancreas cancer*, *to not have a pancreas specific CT protocol is negligent practice” (Radiologist)*
	Cost to the patient	Not having a PPCT or MRI may result in missing out on potential for curative surgery or undergoing unnecessary surgery.	*“Some people will miss out on the potential for a curative operation*, *and others will be operated on unnecessarily*.*” (HPB Surgeon)*
	Routinely applied PPCT or MRI	We do not request or use a PPCT or MRI if it is clear the patient is unsuitable for curative treatment	*“Somebody who is elderly*, *very frail*, *they’ve got a metastatic disease and you’ve done a very poor quality [CT or MRI] but it looks very obvious they've got a metastatic disease; should you put somebody through a CT scan or a MRI scan where that patient it has no bearing on the treatment*. *If they have metastatic disease*, *they are never going to go to surgery*, *they are never going to go through chemotherapy*, *they are elderly*, *and in a nursing home*, *the question would be then why are we doing this*? *The answer is we shouldn’t be doing it in this case*.*” (HPB Surgeon)*
**Motivation and Goals**	Incentives	There are disincentives for undertaking a PPCT or MRI	*“There's a disincentive to do a scan on someone in the private sector*. *If you have a patient who is referred to you as pancreas cancer and they are non-metastatic*, *then there is an opportunity to operate on them*. *If you then do a proper CT and identify that they are unresectable you cannot justify operating on them*. *In the private sector there is potential for unscrupulous activity and not doing the scans and just operating on the patients and finding they are not resectable*. *So*, *it's a bit perverse” (HPB Surgeon)*
		The best incentive is providing good quality care	*“I think the incentive is just patient care*, *I don’t think there's anything more than that*. *And doing the best job by the patient” (Radiologist)*
		Rebates should be offered for MRIs	*“I think it would be ideal if the MRIs were rebated so that you could get them done as necessary in an appropriate facility in a timely manner*. *If a patient’s under surveillance who have a planned scan in 6 months or 12 months they can go to the public hospital and get it done*, *but you still can’t do that for pancreas pathology*, *you can do a magnetic resonance cholangiopancreatography (MRCP) as an outpatient which doesn’t incur cost*. *But the moment you start adding contrast agents into the MRI*, *it costs the patients money*, *and it's a significant amount of money for some people who can’t afford it*. *That needs to be addressed as far as incentive is concerned*, *you need to have it [MRI] accessible so that you can do the appropriate tests at the appropriate time” (HPB Surgeon)*
**Memory, Attention and Decision Processes**	Reminders	There are no reminders in place to prompt us to order a PPCT or MRI	*“No there’s no reminder systems*. *There’re no checklists*. *We just rely on ourselves getting it right*.*” (HPB Surgeon)*
		Multidisciplinary team meetings (MDT) work as a prompt or reminder to order PPCT or MRI	*“If you have missed something*, *that can become quite obvious at the MDT” (Gastroenterologist)*
			*“I mean the MDT process is a safeguard I guess to make sure the patients don’t fall through the cracks in terms of appropriate investigations or tests being performed” (HPB Surgeon)*
**Environmental Context and Resources**	Environmental Stressors	A CT is more accessible than an MRI	*“We have a waiting list for imaging*, *and we have an enormous waiting list for MRI so many months*. *If you are lucky you’d get a CT more than likely next week but you’d get an MRI in many months*.*” (Med Oncologist)*
		Travel is a burden for getting a PPCT or MRI	*“There’s always the tyranny of distance in regional Australia*, *so you know some of my patients have to travel two or three hours by car to get to the closest radiology service*.*” (HPB Surgeon)*
	Rebate or Funders	There are rules or regulations from rebates or funders that influence my decision about using a PPCT or MRI	*“We always make sure we put down MRCP*, *with MRI of the pancreas*, *to make sure there's a decent rebate for the patient” (HPB Surgeon)*
	Costs	PPCT is more affordable than MRIs	*“From the MRI side of things*, *it's a little bit harder because it's a more costly test*, *it's a more intensive test*, *it takes more time*, *and not every patient is suitable*.*” (Radiologist)*
			*“Between normal CT and pancreatic CT there’s little difference but between no MR and an MR there is a huge cost difference*.*” (Radiologist)*
	Organisational Perspective	The volume of cases is a significant consideration on whether the organisation focuses on PC	*“In the post treatment staging and the pre-operative work up that responsibility largely falls to me*. *The reason that has occurred is because I’m the only HPB surgeon here so if I was working at a bigger unit with more HPB surgeons then essentially the arrangement would be different that we would probably have an allied health member who would then become the co-ordinator*, *but we don’t really have that system set up” (HPB Surgeon)*
			*“Our unit here likes to regard itself as a pancreatic centre of excellence*, *I think we perform almost half of the resections across the state*, *we’ve published our outcome results… it's driven a lot by the surgeons*, *but it's a high volume self-declared pancreatic specialist unit” (Radiation Oncologist)*
**Emotions**	Feelings	There can be a nihilistic attitude towards PC	*“There’s a nihilistic attitude*, *even in a meeting that we have for example*, *if the patient’s 83 or 84 and they’ve got pancreas cancer and somebody requests a proper CT*, *there’ll be people who question whether you really want to put them through it*, *the patient’s old and they're not going to get to surgery and you're sort of pushing it*. *So that’s sort of an emotional response rather than a clinical one*, *you know it's a value judgement on the patient and sort of you know rolling your eyes like what's the value of this*, *what's the point of this*?*” (HPB Surgeon)*
		Patients influence decisions on whether to do a PPCT or MRI	*“On occasion patients*, *especially rural patients*, *are very pragmatic and will say I don’t want to know if I have pancreas cancer*, *I really don’t actually want to do that much about it and so there are on occasions some patient factors that might influence your decision about how aggressively to investigate and treat*.*” (HPB Surgeon)*
			*“MRI’s do generate some anxiety in quite a lot of patients and patients often need sedation to have MRI’s*. *They are quite noisy and quite claustrophobic so patients don’t like that*. *If you’re sitting with a patient who needs an MRI and they say “I’ll need to have sedation” you think*, *this means you do generally have to send them to a public hospital because we have less facilities in private centres*.*” (HPB Surgeon)*
**Behavioural Regulation**	Protocols or Referral Pathways	I am not aware of any protocols or referral pathways for PPCT or MRI	*“I think that’s a problem because we don’t follow pathways and we don’t follow a pathway that’s strongly evidence based that embeds quality within it*.*” (Medical Oncologist)*
			*“I’m not sure that it's spelled out in writing anywhere in terms of a protocol*, *you'd have to ask the radiologists that*. *Certainly*, *from a surgical point of view we don’t refer to protocols*.*” (HPB Surgeon)*
			*“I don't know if it's*, *there's a unit handbook that’s given to all the registrars*, *and if they don’t know that they soon find that out*, *because you know you order the scan it's the wrong one you just have to order it again*.*” (HPB Surgeon)*
		We have clear protocols for management of patient with PC	*“We’ve got a pretty coordinated approach to the management of the patients; all the surgeons and oncologists follow the same protocols*. *There has to be good reasons that people wouldn’t have proper treatment*, *we follow pretty aggressive treatment protocols for patients” (HPB Surgeon)*
**Nature of Behaviours**	Established practices	We have well established practices to ensure that the work remains in the vicinity	*“We want to keep the work here and not lose it to metro… why*, *because it's interesting and 2) it serves patients well*. *Part of the integrated cancer work is to provide best work close to home as is reasonably safe” (HPB Surgeon)*

All TDF domains, with the exception of *social influences* were identified by participants as relevant to protocol-based imaging using either a PPCT or MRI, with the domains of *knowledge*, *skills* and *environmental context and resources* being offered by most participants as being relevant in influencing their decisions.

There was variation within the domain of *knowledge* about the differences between a PPCT or MRI and when each modality should be used for diagnosis and/or staging, even between radiologists. Some participants viewed the modalities as equivalent. However, the majority stated that there were clear differences outlining when they would use each. PPCT was viewed as the cornerstone for the staging of PC by the majority of specialists (*beliefs about consequences*) and most believed that if a normal single-phase CT scan or MRI showed clear metastatic disease, then there was no need for further protocol-based imaging (*beliefs about consequences*). Many viewed the MRI as a more challenging modality than a CT scan for a number of reasons. Some specialists described that the MRI required patients to cooperate for a longer period of time, generated anxiety in patients which could require sedation (*emotions*), and half the participants deemed that they were less accessible and more costly to the patient as they did not receive a rebate within the Australian context, *(environmental context and resources)*. While there was a general awareness of some evidence-based clinical practice guidelines relevant to protocol-based imaging, not all were familiar with the recommendations within the guidelines and many did not know of any specific research. A further belief that emerged within this domain was the notion that organisational protocols were more relevant to practice than the guidelines itself (*knowledge*).

A number of surgeons and other specialists stated that they were not aware of, nor did they follow, any protocols or referral pathways to facilitate the implementation of protocol-based imaging. However, those who did follow clear, well laid-out protocols mentioned uniform management across the board, no delays in imaging and a higher volume of surgery undertaken that was concentrated amongst a limited number of surgeons (*behavioural regulation*). Many specialists believed that their organisation had the capacity to manage PC by providing the necessary resources including surgeons, radiologists, oncologists, intensive care and multidisciplinary team (MDT) services. Yet, a number of specialists mentioned that the volume of PC cases determined the focus within the organisation. For example, some described themselves as a “pancreatic centre of excellence” due to the high volumes and focus on PC cases managed within the organisation, whilst a minority alluded to the fact that within their organisations they were the only surgeon managing patients with PC and did not have a specific framework to streamline processes for patients (*environmental context and resources*) which may affect the implementation of a PPCT or MRI.

Overwhelmingly, having access to expert radiologists or radiology practices specialising in pancreatic radiology (i.e. “a good quality radiologist whom you trust”) was viewed as essential for the diagnostic and staging modalities by all craft groups (*skills*). Related to this was the strong belief that quality and interpretation of imaging provided by specialist radiologists play a large role in providing confidence to clinicians in the diagnosis and staging of PC (*beliefs about capabilities*).

It was acknowledged by around half of the participants that the pathway to the diagnosis and staging of PC is complex and can involve numerous disciplines (*social/professional role and identity*). An identified barrier to a timely PPCT or MRI is that referral from primary care to the most relevant specialist (*beliefs about capabilities*) can be intercepted or delayed by referral to other disciplines such as gastroenterology, specialists that may not necessarily undertake a PPCT or MRI as the first step. Conversely, in the metropolitan private sector, a potential barrier identified was the disincentive to undertake full imaging if an opportunity arose to take a patient with potentially resectable cancer straight to surgery (*motivation and goals*).

For all participants, the motivation to undertake a PPCT or MRI was to provide good quality care (*motivation and goals*). However, a belief reported by a minority that emerged as a barrier to PPCT or MRI from the domain of *emotions* was the potential for nihilism, especially in terms of management of older patients who were otherwise well enough to undertake surgical resection. A further belief reported by a few was that in some cases patients themselves were a barrier to optimal care and did not wish to know their diagnosis. In this context imaging may not be undertaken, placing value on the patient perspective (*emotions*).

Related to the patient perspective, a few participants mentioned distance to radiology services as a potential barrier for patients in regional or remote areas, while others recognised that often these patients were resigned to the fact that they would have to travel. Whether these decisions were overlayed as a consequence of patients’ preference or a result of clinical therapeutic nihilism is difficult to estimate (*environmental context and resources*). Related to patients with PC living in regional areas was the belief that “having a succession plan, an age range of clinicians… with a policy of working together”, and establishing sound practices, was a driving force that would enable services to remain in the regional vicinity and not be lost to the cities (*nature of behaviours*). It was further highlighted by regional specialists that a quality PPCT would detect the more complex cases such as those needing vascular resections, and these would then need to be referred to surgeons based in the metropolitan areas with expertise in this type of resection.

## Discussion

Appropriate implementation of a PPCT or MRI reduces the risk of an adverse outcome in patients who are diagnosed with non-metastatic PC. Proper staging including PPCT/MRI allied with assessment of co-morbidities enables informed personalisation of therapy to a surgery first, neoadjuvant, chemotherapy or supportive pathway, minimising futile interventions. Behavioural research can help identify the factors that influence the implementation of this best practice. To our knowledge this is the first study to explore the barriers and enablers to the use of PPCT or MRI for diagnosis and/or staging in PC using the TDF as a guiding framework. The use of the TDF allowed a comprehensive evaluation of specialists’ behaviour with respect to this practice, using a systematic approach. It provides a basis from which to tailor future interventions aiming to overcome barriers and harness enablers, improving uptake of PPCT or MRI and therefore potentially improving outcomes for patients diagnosed with PC. Further, our approach to this study was based on methods implemented by Graco and colleagues which have been important in categorising the opinions of specialists [23]. However, in our study we opted not to include the frequency of belief statements to avoid important beliefs being overlooked.

Current practice shows that diagnosis of PC is often reliant on preliminary imaging such as a single-phase CT scan or an endoscopic ultrasound (EUS), followed by referral to appropriate specialists who may then order a PPCT to stage PC preoperatively if appropriate (S1 Fig). *Knowledge and skills* were revealed as important domains in this study, as it was not widely understood that there is a hierarchy approach to the application of PPCT or MRI. We did not anticipate this gap in knowledge or awareness related to the recommendations within clinical practice guidelines and the differences in perspectives on the information provided by a PPCT compared to MRI. The PPCT has higher accuracy in identifying the extent of PC, locoregional extension, vascular invasion, distant metastases and resectability, whereas the MRI has some advantages over CT in detecting small tumours, distant metastases, especially to the liver, and isoattenuating tumours [24]. Therefore, MRI is best reserved for diagnosis rather than staging of PC, particularly as highlighted in our study, it is a more challenging modality both from a clinical and patient perspective.

Pre-operative staging using a PPCT, ideally reported using standardised templates, provides confidence and enhances surgeons’ ability to better select patients for surgical resection [25]. A practice of concern was the motivation to undertake surgery without the necessary preoperative staging by some surgeons. Without proper staging using a PPCT, the likelihood of achieving complete curative resection is at risk and patients who may have benefited from neo-adjuvant therapy may miss out due to surgery undertaken too early [1]. Organisations, should have systems in place to ensure patients with clinical suspicion of PC undergo proper staging to better select patients likely to benefit from surgery with curative intent. A potential safeguard is the mandatory presentation of patients to MDT meetings prior to management decisions being reached, especially where considerable multidisciplinary expertise is essential for optimal care and all the more so in smaller/low volume centres [26]. The use of protocols and algorithms for investigation and staging will be more likely to ensure uniformity and equity of access to care [27].

Although not viewed as an impediment to receiving a PPCT or MRI, several factors resulted in delays to receiving appropriate imaging. Such factors included referrals to other specialists, the distance travelled to reach radiological services, as well as the technical details of the actual scans, accessibility of the reports and finally the quality of the scan and interpretation of the radiological report. A cross-sectional study assessing presentations to general practitioners’ (GP) before a cancer diagnosis found that PC was associated with the highest risk of presenting multiple times before being referred to an appropriate specialist [28]. Further research is required to understand the timeliness of referrals, the impact of distance on protocol-based imaging and the quality of imaging in primary care. Participants in this study believed that radiologists and radiology services that specialise in pancreatic radiology produce imaging and reports of higher quality.

An opportunity also exists for radiologists to play a more proactive role in contacting the primary provider to hasten the implementation of a PPCT in a single session if they become suspicious of pancreatic aetiology. Therefore, further research would explore: (1) the GP’s understanding of the importance of referring patients to specialist radiology services; and (2) radiology centres taking responsibility for ensuring that patients with a suspicion of PC receive the best available service (requiring the necessary expertise in pancreas radiology).

Within the domain of *environmental context and resources*, it was widely understood that without access to PPCT and skilled radiologists, hospitals with a low volume of cases may be disadvantaged compared to high-volume tertiary metropolitan-based centres. A meta-analysis reviewing the relationship between hospital volume and outcome confirmed the association between higher hospital volume and lower post-operative morality and lower length of stay [29].

This study provides detailed insights into the perceptions of specialists involved in deciding whether a patient has a PPCT or MRI and whether it is undertaken. The breadth of the specialists involved in the project across two states in Australia provides a novel and broad view of barriers and facilitators.

Our study had some limitations. Radiologists who had agreed to participate in the focus group were unable to attend on the day of the meeting. We had less participation from disciplines other than surgeons in NSW, and this limits our ability to report on a range of perspectives from this state. In a similar respect we may not have captured all perspectives from individual disciplines due to lower participation. For example, only one radiation oncologist was interviewed in this study. This may bias the findings of our study. Further, a well-known concept in qualitative research is data saturation. We interviewed a heterogenous population. However, data was collected until no new information presented on the barriers and enablers within the 12 TDF domains [30].

### Recommendations

Our study highlights the importance of the TDF domains of *knowledge*, *skills* and *environmental context and resources* in understanding the diagnosis and staging practices for PC.

Clinical practice guidelines that are often systematically developed, based on the most current evidence, have the potential to improve health outcomes and provide consistency of care [31]. Deviation from guidelines can be a perceived barrier if organisational protocols or procedures do not align with international, peer-reviewed, evidence-based guidelines. Organisational protocols should also be revised and kept up-to-date with current evidence [32]. Given that a barrier to the routine implementation of PPCT is the lack of knowledge, we recommend the dissemination of knowledge to clinicians, and mechanisms to profile such guidelines through forums that provide educational opportunities.

There is evidence that audit and feedback, and reminders can attain positive behaviour change [33]. Further, an earlier review highlighted a lack of health services research examining the influence of guidelines in PC management [34]. This study adds important literature on the use of clinical pathways to facilitate the implementation of guidelines by identifying practices and beliefs that prevent evidence-based care. An important consideration is the role of MDT meetings as a reminder for undertaking PPCT or MRIs, providing access to a range of relevant disciplines, especially specialist radiologists and a forum for receiving feedback. While MDT meetings are endorsed as best practice in cancer care, they are not mandatory and this can limit the consistency in care received by patients [35].

## Conclusions

It is imperative that diagnosis and staging investigations using the most appropriate imaging modalities are conducted in a timely, efficient and effective manner. This qualitative study used a knowledge translation approach and psychological theories in health to explore factors associated with implementing the appropriate imaging investigations for diagnosis and/or staging in PC. The results provide an understanding of specialists’ opinion and behaviour in relation to a PPCT or MRI and should be used to inform the design of future interventions to improve compliance with this practice.

## Supporting information

S1 File Interview schedule. (DOCX)

S1 Fig A decision tree for protocol imaging and multidisciplinary team meetings for patients diagnosed with pancreatic cancer. (TIF)
