# Supplementary material for: Barriers and enablers to the implementation of protocol-based imaging in pancreatic cancer: A qualitative study using the theoretical domains framework
Source: PLoS One. 2020 Dec 17;15(12):e0243312. doi: 10.1371/journal.pone.0243312 (PMC7746147; doi:10.1371/journal.pone.0243312)
Supplement: S1 File — (DOCX) [file pone.0243312.s001.docx]

**S1 File. Interview Schedule (PPCT/MRI)**

**TITLE:** Barriers and Enablers to Selected Indicator Implementation in Pancreatic Cancer: a qualitative study using the theoretical domains framework.

**Introduction**

As outlined in the explanatory statement, this study aims to identify and explore the barriers and enablers to:

1. Implementation of a PPCT or MRI scan for diagnosis and/or staging

- We are interviewing a range of specialists across Victoria and NSW, who manage patients with pancreatic cancer, particularly in relation to the two practices we are exploring
- Interviews will be recorded
- You can stop the interview at any time
- We are using a framework known as the theoretical domains framework that include 12 domains. Some questions may appear repetitive but the approach is to follow a structure so that we don’t miss any important concepts and we understand from your perspective how these practices work in the real world.
- This study will be published and data will be aggregated. We can assure you that no identifiable data will be published.

Before we start do you have any questions?

**Background information**

Can you describe your role and area of care?

Prompt questions

- How many years of experience do you have working as a surgeon/ oncologist/ radiologist/ chair of a MDT / care coordinator?
- How many cases of PC would you see in a week?
- Do you work in the private or public sector or both?
- Do you work mainly in metropolitan areas, regional or both?
- What is the mix of resectable vs non-resectable PC cases you see?
- To understand some of the systems in place within your organization: What type of medical record system is in use? Paper based / electronic? Use of reminders/prompts/pathways in system?
- Is there access to a radiology department in your hospital? What radiology services do they offer? If not, where are the nearest radiology services? Is there a waiting list for services? (can potentially be under the domain environmental context?)

Earlier a decision tree that outlined the potential process incorporating protocol imaging was circulated. Are there any comments regarding the decision tree?

Now I am going to ask you questions on the factors that influence the practice we are studying.

**Implementation of a PPCT or MRI scan for diagnosis and/or staging (Surgeons/Oncologists /Radiologists)**

**Understanding clinical practice**

Prompt questions:

- Are you involved in the ordering or interpreting of the results of the diagnostic tests PPCT or MRI for suspected PC?
  - If so, what tests do you order? How would you decide which test between the two?
  - Do you believe the tests are equivalent?
  - Which patients do you test for PC?
  - Roughly what proportion of your HPB patients do you refer for testing for PC?
  - Where are the results of the assessments/tests recorded? (and by who?)
  - Do you know roughly how long your patients wait for a diagnostic test?

**Factors influencing practice**

In the Theoretical Domains Framework, there is a set of 12 domains that are known to influence clinical behaviors.

Firstly, before I prompt with specific questions about potential factors that are known to influence clinical practice, can I first ask you what you think are the biggest influences on your decision to order or use a PPCT or MRI for patients with a clinical suspicion of PC in your hospital?

Prompt questions to explore factors influencing practice (grouped by TDF domains).

| **TDF Domains** | **TDF Definitions [Constructs]** | **Prompt questions** |
| --- | --- | --- |
| Thinking about Knowledge | An awareness of the existence of something. [Knowledge including knowledge of condition/scientific rationale. Procedural knowledge. Knowledge of task environment.] | **Are you aware of any clinical practice guidelines on the management of patients with clinical suspicion/diagnosis of PC?** Do you feel your colleagues are aware of these guidelines?  How credible are the sources or developers of these guidelines?  **Are you familiar with the recommendations in these guidelines for referral for imaging?**  Are you aware of any protocols within your organization for CT scans or MRIs in the diagnosing/staging pancreatic cancer?  Were they externally / internally developed?  What do you think is best practice in the diagnosis and staging of PC?  **Are you aware of any research about the prevalence and impact of PPCT or MRI on the diagnosis and staging of PC?**  **When you order a PPCT what do you expect a radiologist to do?** |
| Next we want to understand the Skills that maybe required | An ability or proficiency acquired through practice. [Skills Skills development Competence Ability Interpersonal skills Practice Skill assessment] | **Apart from radiology, What skill sets are needed to use of a PPCT or MRI?**  How would you go about ordering the test?  Are these scans difficult to interpret? |
| Now thinking about Social professional role and identity | A coherent set of behaviors and displayed personal qualities of an individual in a social or work setting. [Professional identity Professional role Social identity Identity Professional boundaries Professional confidence Group identity Leadership Organizational commitment] | **In addition to your role in diagnosing or staging PC, who else maybe involved in diagnosing and staging?**  **Thinking about this from an organizational perspective, could you describe the focus within your organization to manage PC?** Or is the management of PC referred to other centres (e.g. metropolitan hospitals) |
| Moving onto the domain Beliefs about capabilities | Acceptance of the truth, reality, or validity about an ability, talent, or facility that a person can put to constructive use. [Self-confidence Perceived competence Self-efficacy Perceived behavioral control Beliefs Self-esteem Empowerment Professional confidence] | Any difficulties in assessing the results from a PPCT or MRI?  **What are the challenges in determining/ diagnosing the presence of PC in general and using/ordering different tests/tools in particular?**  What would help you to identify your patients with PC?  **How confident would you and your peers be that you can identify PC in your patients through the implementation of a PPCT or MRI?** |
| Now thinking about the Beliefs about consequences | Acceptance of the truth, reality, or validity about outcomes of a behaviour in a given situation. [Beliefs Outcome expectancies Characteristics of outcome expectancies Anticipated regret Consequences] | **What do you think your peers or colleagues believe are the benefits and costs of implementing a PPCT or MRI? (for your patients, you, your colleagues and the organization)**  What are the benefits and costs of not implementing a PPCT or MRI? (for your patients, you, your colleagues and the organization)  **Do you or your peers believe there are any consequences for not routinely applying a PPCT or MRI where there is suspicion of a pancreatic lesion?**  Do you think the benefits of these tests outweigh the costs?  Does the evidence suggest that implementing a PPCT or MRI is worthwhile? |
| Coming now to Motivation and goals | A conscious decision to perform a behavior or resolve to act in a certain way. Mental representations of outcomes or end states that an individual wants to achieve. [Stability of intentions Stages of change model Transtheoretical model and stages of change Goals (distal/proximal) Goal priority Goal/target setting Goals (autonomous/controlled) Action planning Implementation intention] | **Should there be incentives to implementing a PPCT or MRI for all suspected cases of PC?**  What could these be?  What would be the implications of not having adequate imaging?  **Are there occasions where your peers and colleagues feel there is no need to order a PPCT or MRI before any treatment**  Are there other aspects of your role that interfere with the implementation of a PPCT or MRI for all suspected cases of PC? |
| We are now going to discuss Memory, attention and decision processes | The ability to retain information, focus selectively on aspects of the environment and choose between two or more alternatives. [Memory Attention Attention control Decision making Cognitive overload/tiredness] | **Are there any reminders in place to prompt you and your colleagues to do any of the relevant tests? If not, do you think these would be helpful?’**  Is ordering a PPCT or MRI something you do routinely for clinical suspicion of PC? |
| Thinking now in terms of the Environmental context and resources | Any circumstance of a person’s situation or environment that discourages or encourages the development of skills and abilities, independence, social competence, and adaptive behavior. [Environmental stressors Resources/material resources Organizational culture/climate Salient events/critical incidents Person x environment interaction Barriers and facilitators] | **Are there any resources that influence whether you or your peers order a PPCT or MRI?**  Are there sufficient human resources?  Are there sufficient physical resources?  Do you have enough time/do you have competing demands?  Does the working environment have an effect?  **Are there environmental stressors that impact on the ability for you and your peers to implement a PPCT or MRI?**  **Are there rules/regulations from rebate or funders that influence the decisions about using a PPCT or MRI for diagnosis or staging?** |
| Thinking about Social influences | Those interpersonal processes that can cause individuals to change their thoughts, feelings, or behaviors. [Social pressure Social norms Group conformity Social comparisons Group norms Social support Power Intergroup conflict Alienation Group identity Modeling] | Do you seek opinions of colleagues in whether to use a PPCT or MRI in suspected cases of PC?  **What are the views of your colleagues regarding the use of a PPCT or MRI routinely?** |
| The next domain in the framework is centred on any Emotions attached to this practice | A complex reaction pattern, involving experiential, behavioral, and psychological elements, by which an individual attempt to deal with a personally significant matter or event. [Fear  Anxiety Affect Stress Depression Positive/negative effect Burn-out] | **How do you and your colleagues feel about ordering a PPCT or MRI for all patients with suspicion of PC?**  Does this alter your clinical management decisions? |
| Finally, on Behavioral regulation | Anything aimed at managing or changing objectively observed or measured actions. [Self-monitoring Breaking habit Action planning] | **Are there any protocols or referral pathways that facilitate the implementation of a PPCT or MRI scan for patients with suspicion of PC?** |

***Bolded statements –*** *prompts used often*

If time permits and only if these items have not yet been covered:

- What do you think are the key actions/decisions when managing a patient with PC that maximize the beneficial outcomes for the patient in relation to these two practices?
- Is there an aspect of the patient pathway we should pay more attention to in future interviews?
- If there was one thing you could change in your hospital to improve the management of patients with PC, what would you change?

Final questions:

- Is there anything else about the management of patients with PC that you would like to mention that is not already covered?
- Do you have any additional comments on the content of the interview or feedback on how the interview went?

**THANK YOU VERY MUCH FOR YOUR TIME**
