# Supplementary figures and images for: Barriers and enablers to the implementation of protocol-based imaging in pancreatic cancer: A qualitative study using the theoretical domains framework
Source: PLoS One. 2020 Dec 17;15(12):e0243312. doi: 10.1371/journal.pone.0243312 (PMC7746147; doi:10.1371/journal.pone.0243312)

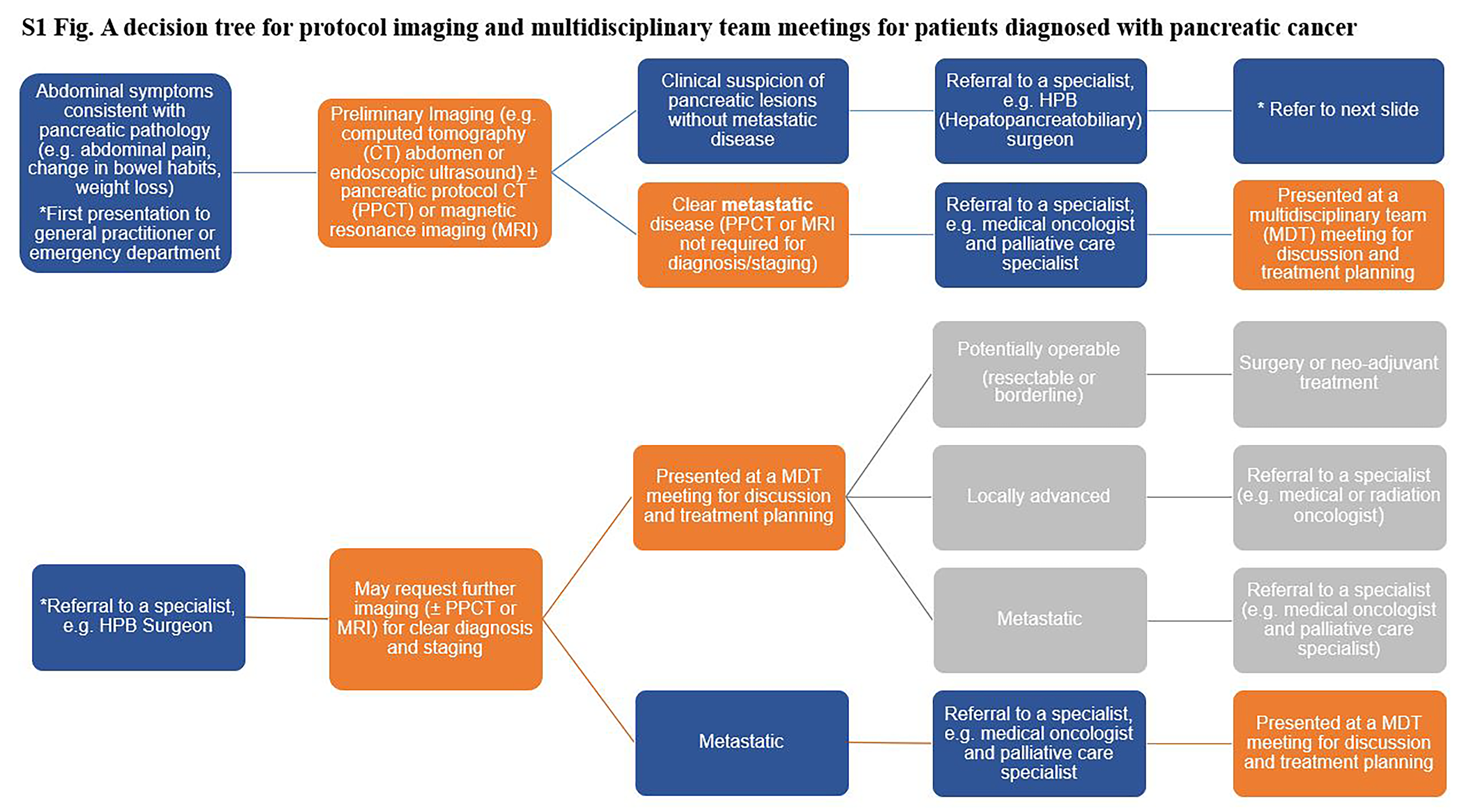

Supplement: S1 Fig — (TIF) [file pone.0243312.s002.tif]
